# Equipping community health workers in Rwanda to deliver a gender transformative parenting program to prevent violence against women and children at scale

**DOI:** 10.3389/frph.2025.1602136

**Published:** 2025-06-23

**Authors:** Kate Doyle, Isha Bhatnagar, Emmanuel Karamage, Jean Paul Tuyisingize, Chantal Muhimpundu, Ange Marie Yvette Nyiransabimana, François Regis Cyiza, Fidèle Rutayisire, Silas Ngayaboshya, Webster Mavhu

**Affiliations:** ^1^ Department of Research, Evaluation & Learning, Equimundo: Center for Masculinities and Social Justice, Washington, DC, United States; ^2^ Department for Public Health and Primary Care, Ghent University, Ghent, Belgium; ^3^ Rwanda Men’s Resource Center, Kigali, Rwanda; ^4^ Division of Maternal Child and Community Health, Rwanda Biomedical Center, Kigali, Rwanda; ^5^ Directorate of Gender Promotion and Women Empowerment, Ministry of Gender and Family Promotion, Kigali, Rwanda; ^6^ Department of SRH & MNCH, Centre for Sexual Health & HIV/AIDS Research Zimbabwe (CeSHHAR), Harare, Zimbabwe; ^7^ Department of International Public Health, Liverpool School of Tropical Medicine, Liverpool, United Kingdom

**Keywords:** intimate partner violence (IPV), gender transformative, community health workers (CHWs), health system, scale, parenting, fathers, Rwanda

## Abstract

**Introduction:**

In Rwanda, the Bandebereho program has demonstrated long-term reductions in intimate partner violence (IPV) and violence against children. Since 2019, the program has partnered with government to train community health workers (CHWs) to deliver at scale. Evidence on how to equip CHWs to deliver Bandebereho, or similar programs, with quality and fidelity is needed to support scaling. This study sought to assess the impact of training on CHWs and their capacity to deliver Bandebereho during scale up.

**Methods:**

A pre/post, follow-up study was conducted with 573 CHWs in Burera district. Data were collected at three time points over 20 months using self-administered questionnaires (pre/post) and a follow-up phone survey. Questionnaires gathered data on CHW attitudes about gender roles and violence, self-reported skills, knowledge and confidence to implement Bandebereho, and training impacts on partner relations and community work. Informed consent was obtained from all study participants.

**Results:**

The pre-survey was completed by 562 CHWs and 564 CHWs completed the post-survey after six to nine months. The phone survey was administered to 506 CHWs at follow-up (at 17–19 months). Analysis of changes between pre- and post-surveys found CHWs had more equitable gender attitudes after the training. Linear regression analysis found that CHWs with some secondary education (coefficient: −2.15, *p* < 0.01) and more than three years' experience (coefficient: −2.27, *p* < 0.001) were less likely to hold inequitable attitudes. At post-survey, CHWs reported a high level of preparedness to implement, regardless of gender. A majority reported improved partner relations, including greater partner support for their community work. At follow-up, a majority of CHWs reported a high degree of comfort and confidence implementing Bandebereho, and benefits to their work and personal relationships.

**Conclusions:**

The findings highlight the importance of investing in high-quality facilitator training, which allows sufficient time for facilitators' own transformation, to maintain quality and fidelity at scale. The findings underscore the importance of a slow and steady approach, with sufficient time to adapt, test, and refine IPV programs for scale, which can also support a progressive handover to government. The findings may support program originators who seek to scale proven IPV prevention programs with government in other settings.

## Introduction

Intimate partner violence (IPV), the most common form of violence women experience globally, is preventable (1). Rigorous evidence indicates programs can prevent or reduce IPV within three to five years, including time for development and testing prior to implementation (2, 3). Gender transformative programs that work with groups of men and women to promote critical reflection on harmful gender norms and unequal power dynamics, and build relationship skills like communication and conflict resolution, are a common approach (4, 5). Yet many programs never move beyond small-scale pilots, hindering our ability to reduce violence at the population level (6). Proven programs need to be scaled up to expand their reach, impact, and sustainability (6–9). One strategy is to partner with governments to embed and deliver programs through the public sector, also called institutionalization or vertical scaling (10–12). Although complex and challenging, public sector scaling can make programs more accessible, equitable and affordable (10–15). This strategy is often used for scaling sexual and reproductive health programs for example, but is relatively underutilized for IPV prevention outside of educations systems (6, 14).

Public health systems are a potential pathway to scale IPV prevention programs, given their reach and frequent interactions with women, including during life stages characterized by increased risk of violence. Many countries rely on community health workers (CHWs) to provide frontline health services. CHWs are often lay workers who receive limited training, and are trusted community members who interact daily with women and families (16). Thus, CHWs are well placed to support IPV prevention (17–19), and it makes sense given the direct consequences such violence has on women's and children's physical and mental health (1). The inequitable gender norms that perpetuate violence also influence women's and children's care-seeking and health outcomes, which is why many gender transformative programs seek to prevent IPV and to promote maternal or child health, family planning, or HIV prevention (20, 21). Emerging evidence indicates that CHWs can facilitate these programs, as demonstrated by pilots in Niger and Tanzania that reduced IPV and improved health outcomes for women and children (22, 23). Working with CHWs to deliver such programs at scale could provide a cost-effective way for governments to achieve select health and development goals.

### The Bandebereho program and context

In Rwanda, the Bandebereho (“role model”) program is scaling up by training CHWs to deliver the approach in their communities. Bandebereho uses fatherhood as an entry-point to engage men and encourage caring and non-violent relationships with their partners and children. Men who are expectant or current parents of young children are invited to participate, alongside their partners, in small group sessions of critical reflection, discussion, and skills building (20). A structured curriculum promotes reproductive, maternal, newborn, and child health, men's caregiving, and healthier couple relations. The theory of change proposes that creating a safe space for men and their partners to reflect on the costs of rigid gender norms and to learn and practice more equitable attitudes and relationship skills can lead to positive changes across a range of health and relationship behaviors. Bandebereho aligns closely with Rwanda's national reproductive, maternal, newborn, and child health (RMNCH) policies and strategies, including its 2030 health goals to ensure all pregnant women receive sufficient and timely antenatal care and to address any unmet need for family planning (24–26). The country's health, gender, and early childhood development policies acknowledge the need to engage men and to challenge harmful gender norms, including to prevent violence and support women's and children's care-seeking (25–28). Despite strong laws and policies, unequal gender norms and high rates of intimate partner violence persist. Most recent national data indicate that 46% of ever-married women have experienced physical, sexual, or emotional IPV from their current or most recent partner (30% in the past year), an increase from the previous five years (29, 30). Attitudes accepting of violence have also risen, with 50% of Rwandan women and 18% of men believing that a husband is justified in beating his wife in at least one of five circumstances (29).

Bandebereho was piloted from 2013–2015 by the Rwanda Men's Resource Center (RWAMREC) and Equimundo, in collaboration with the Rwanda Biomedical Center (RBC), which implements Rwanda's health policies. RWAMREC, a national non-governmental organization that engages men as allies in gender equality, trained carefully selected fathers to facilitate the 15-session curriculum in their communities. The pilot, which reached more than 3,000 parents, was highly attended and strongly resonated with participants (31, 32). A randomized controlled trial (RCT) demonstrated substantially lower rates of physical, sexual, economic, and moderate or severe emotional IPV among female participants compared to a control group at 21 months (32). Parents also reported lower rates of violence against children. These impacts were sustained at six-year follow-up, alongside lasting improvements in women's antenatal care seeking and men's accompaniment, parental depression, child behavioral outcomes, and more equitable patterns of household caregiving and decision-making (20, 33). Structural equation modelling found that no one component drove the reductions in IPV, although more positive couple dynamics, including more frequent communication and emotional closeness, accounted for the largest share of the program's effect, followed by changes in men's attitudes about gender (34).

### The scaling pathway

The success of the pilot led to calls to scale Bandebereho to reach more Rwandan families (35). Scaling through the health system made sense given RBC's longstanding involvement and Bandebereho's alignment with national RMNCH policies. CHWs were chosen given their countrywide presence, role in promoting RMNCH, and relative stability compared to other community structures, including local gender-based violence and child protection mechanisms. Training CHWs to implement Bandebereho also aligned with Rwanda's efforts to expand health service access through task-shifting to CHWs and task-sharing with other community actors, which complement efforts to increase the number of skilled providers. Rwanda's CHWs are elected volunteers trained to provide basic health information and services at the village level. An integral part of the decentralized health workforce, CHWs receive some compensation through a performance-based financing system and participation in income-generating cooperatives (16). Recent reforms have shifted away from CHWs with specialized roles toward a polyvalent model where all CHWs are being trained to provide a comprehensive integrated health package.

The vision of scale is to make Bandebereho accessible to parents in all 30 districts by integrating Bandebereho delivery in the health system, including program training, implementation, supervision, and monitoring, with tapering support from RWAMREC (36). Currently, Bandebereho is being scaled from one district to three, through a phased approach which allows for careful adaptation, testing, and refinement of the program for health system integration (13, 14). This gradual approach was chosen to overcome the common challenges program originators face in maintaining fidelity and quality at scale (6, 8, 37, 38), which are essential for sustaining program impact (39, 40). Since 2019, RWAMREC has worked with RBC to adapt Bandebereho for delivery by CHWs, overseen by a multisectoral advisory group including the Ministry of Gender and Family Promotion and the National Child Development Agency. The scaling process began with the transition-to-scale phase (2019–2022), where RWAMREC trained CHWs in one district to implement Bandebereho. Careful supervision and monitoring was done by RWAMREC to assess implementation fidelity and quality, meaning adherence to the program's core components (e.g., recruitment, content, frequency, duration) and principles (e.g., participant interaction and voice), respectively (40–42). The current scale up phase (2023–2026) seeks to further optimize the program for health system integration through iterative cycles of adaptation and testing as the program expands from one to three districts. The learning generated will inform the development of a national scale up strategy.

This article presents findings and lessons learned from training CHWs to deliver Bandebereho. Facilitators of gender transformative programs like Bandebereho play a key role in supporting participants through a process of change (37, 38). They must be able to foster open, nonjudgmental discussion that can prompt group participants to question or challenge the persistence of gender norms that promote men's dominance over women or justify men's use of violence against women (37, 43). Being able to motivate group members to adopt the positive behaviors being promoted requires facilitators to first internalize the changes they seek to effect in others (37). High-quality training and supervision are therefore critical to enable facilitators to reflect on their own attitudes and behavior, develop participatory facilitation skills, and acquire the skills and confidence to promote more equitable relationship behaviors. Working with government requires adapting training and supervision to meet the needs of the existing workforce (13–15), in this case CHWs, rather than the carefully chosen facilitators who typically deliver IPV prevention programs (4, 43). Further, program originators like RWAMREC often face pressure to reduce costs by shortening program training or duration when scaling (6–8). These challenges can hinder implementation and the potential to sustain impact at scale. In scaling Bandebereho, RWAMREC set out to test different training models to generate evidence on what works to train CHWs to deliver Bandebereho. Our study assessed the facilitator training model used in the current scale up and its impacts on CHWs and their capacity to effectively deliver Bandebereho, to inform future scaling.

## Materials and methods

### Study setting and design

The study was conducted with 573 CHWs from all 571 villages in Burera, a mostly rural district in Rwanda's Northern province, between June 2023 and January 2025. Bandebereho was first adapted for delivery by CHWs in neighboring Musanze, a semi-urban district in the same province, during the transition-to-scale. Burera was selected as the second district due to: its proximity to Musanze (allowing for staffing and cost efficiencies); buy-in from district and provincial leadership; and the recommendation of the technical advisory group, given the district's high rates of IPV, malnutrition and stunting (29). Ensuring equity in access to services was also a consideration, as Burera is relatively underserved by social and behavior change programs given its remoteness and limited accessibility. The district's population of nearly 400,000 is organized within 17 administrative sectors, 69 cells, and 571 villages (44).

**Figure 1:**
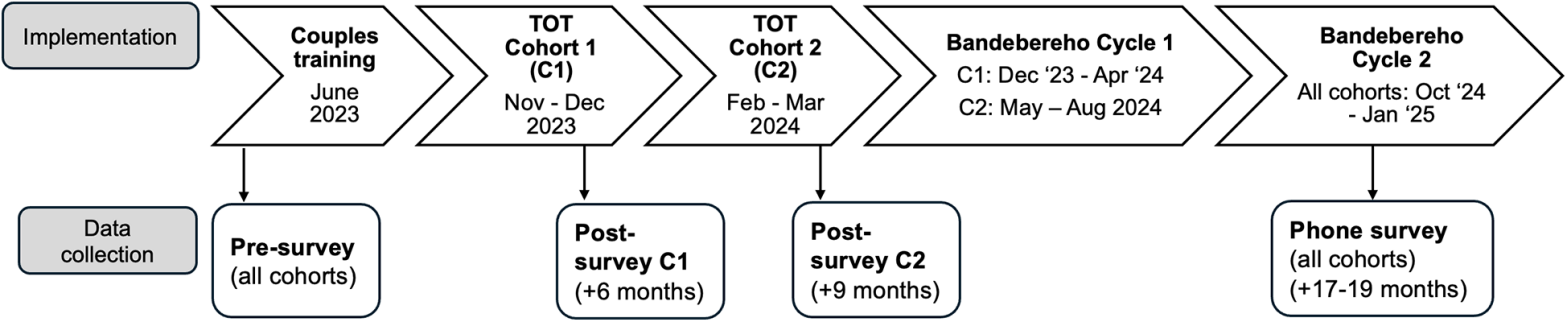
Implementation and data collection timeline.

The study used a pre/post follow-up design to assess the impact of an adapted CHW training model over a 20-month period. This design was chosen to assess the effects of the training over time, and to capture CHW experiences of both training and program implementation, while minimizing research costs to support implementation and monitoring. Data were collected from CHWs at three time points: (1) *pre-survey* before initiating training (June 2023); (2) *post-survey* after completing training (December 2023 or March 2024, six to nine months after pre-survey); and a (3) *follow-up* phone survey during program implementation (November 2024 to January 2025, 17–19 months after pre-survey). Routine monitoring data were analyzed to assess CHW participation in training and the fidelity and quality of Bandebereho implementation by the trained CHWs. Figure 1 illustrates the implementation and data collection timeline.

### Study population

Our study sought to collect data from all 573 CHWs (277 female, 296 male) trained to deliver Bandebereho in Burera district. Rwanda recently began shifting to a polyvalent model where all CHWs will deliver an integrated package of health services. Prior to this change, each village in Rwanda had up to four CHWs with specialized roles: *animatrices de santé maternelle* (ASM) responsible for supporting pregnant women, including accompanying them to antenatal care visits; *binômes*, a male-female pair responsible for a range of early diagnostic and treatment services for maternal and child health; and more recently, a health promotion and disease control CHW (male or female).

During the transition-to-scale RWAMREC worked nearly exclusively with the health promotion CHWs to deliver Bandebereho in Musanze district. At the time, RWAMREC and RBC selected the health promotion CHWs given their recent election and lower workload compared to other CHWs. Two health promotion CHWs were paired together to co-facilitate (one male and one female where possible) a Bandebereho group, whereas one male peer educator facilitated a group in the pilot. The Musanze experience indicated the majority of CHWs could deliver Bandebereho sessions with fidelity and quality when trained directly by RWAMREC. However, low literacy levels and limited motivation hindered some CHWs; they were assigned a supporting role alongside a pair of stronger CHWs.

In the current scale up in Burera, the site of our study, CHWs were selected according to eligibility criteria designed to overcome the literacy and motivation challenges observed in the transition-to-scale. While seeking roughly equal numbers of female and male CHWs, RWAMREC and health authorities chose at least one CHW per village who: (i) had completed at least primary school; (ii) was ideally 25–50 years old; (iii) did not hold multiple community roles (e.g., being a CHW and a village leader or public servant) which might limit availability; (iv) was a stable resident; and (v) was motivated to be involved. This change in selection also aligned with Rwanda's ongoing transition to a polyvalent CHW model.

### Bandebereho training for CHWs

Several adaptations to the training model were made in the scale up phase assessed in our study, including: (i) new criteria for selecting CHWs; (ii) expanded training time and components; (iii) greater health system leadership in conducting training; and (iv) introduction of remuneration for CHWs facilitating Bandebereho. CHWs in Burera received two distinct trainings designed to equip them with the knowledge and skills to facilitate an extended 17-session version of the Bandebereho curriculum, which includes more focus on maternal and child nutrition and unpaid care work. RWAMREC tailored the training to meet the varying capacities of the existing workforce, which had no prior experience facilitating participatory and interactive sessions.

CHWs first participated in a four-day gender transformative couples training with their spouse or intimate partner in June 2023. Nearly all CHWs (98%) and their partners attended the full training. The training, a condensed version of the Bandebereho curriculum, aimed to: promote critical reflection of unequal gender norms and power dynamics; increase couple communication, shared decision-making, and men's participation in unpaid care work; and build trust and mutual support between partners. The training used participatory activities, such as role plays, drawing, case studies, and small group discussion. Topics included, for example, the difference between gender and sex, understanding forms of violence, disrupting intergenerational patterns, sharing household responsibilities, making a household budget, and alcohol and drug abuse (a risk factor for IPV). The training also created space for CHWs to discuss their community work with their partners.

The couples training was an integral part of facilitator training in the Bandebereho pilot. In the transition-to-scale, the training was initially removed to reduce CHW training time and costs. However, RWAMREC staff observed that CHWs reported high levels of mistrust, conflict, and violence in their relationships. Subsequent research commissioned by RWAMREC found that couple conflict often hindered the work of CHWs, particularly females (45). RWAMREC recommended reintroducing the couples training and raised funding to provide it, but it was conducted after CHWs in Musanze had begun implementing Bandebereho. Based on that learning, the couples training was planned as the first of two trainings for CHWs in the scale up in Burera district.

After the couples training, CHWs in our study received a 15-day training of trainers (TOT) that included eight days of theory and seven days of practice-based learning designed to build their knowledge, skills, and confidence to facilitate the Bandebereho curriculum. The training was divided into three five-day trainings, delivered every other week over a five-week period. The theory portion focused on how to use the curriculum, the session structure (i.e., check-in, participatory activities, group discussion, closing), participant selection, monitoring and reporting guidelines, and observing the sessions facilitated by trainers. The practice portion focused on building CHWs' facilitation skills, practicing co-facilitating up to seven (out of 17) of the most challenging sessions in the curriculum, and receiving constructive feedback from peers and trainers. On off weeks, CHWs performed their normal duties and met with village leaders to prepare Bandebereho recruitment and implementation. Emerging recruitment challenges were troubleshooted in the TOT. Initially planned for ten days (as in the transition-to-scale) the TOT was extended to fifteen days—due in part to an unforeseen months-long delay between the couples training and TOT, which necessitated refresher on content covered in the first training.

Separate TOTs were organized at each health center to train CHWs working in the catchment area. Health centers with larger numbers of CHWs were subdivided to cap participants at 35. In total, 27 trainings were held (with 21 participants on average). The TOTs were staggered in two cohorts. Cohort 1 (302 CHWs from ten health centers) completed training in December 2023 and Cohort 2 (271 CHWs from nine health centers) in March 2024. Nearly all CHWs (99%) attended the TOT in full. After completing the first cohort, RWAMREC staff observed that some CHWs still had relatively weak facilitation skills and some CHWs asked for more practice time. RWAMREC modified the training for the second cohort to enable more practice time. By increasing the number of trainers and training rooms and subdividing participants, more CHWs were able to practice simultaneously. As a result, CHWs in Cohort 2 practiced up to seven sessions, while Cohort 1 managed only four. A second change was to conduct a meeting between CHWs and local leaders during rather than after the TOT. This meeting was convened by the Executive Secretary of each cell (a subdistrict administrative unit) to facilitate communication and support Bandebereho implementation.

In the transition-to-scale, RWAMREC led the training, supervision and monitoring of Bandebereho by CHWs. In the current scale up, Community Environmental Health Officers who are based at local health centers and oversee CHWs in their catchment area (hereafter referred to as CHW supervisors) co-facilitated the couples training with RWAMREC and led the TOTs. Seventeen CHW supervisors from Musanze district with multiple years of experience with the Bandebereho program acted as lead trainers. They were paired with 19 CHW supervisors from Burera district, who received a four-day gender transformative training and a five-day TOT co-led by RWAMREC staff and the Musanze CHW supervisors. Supervisors from Musanze received similar training in 2019 and additional orientation from RWAMREC prior to the Burera trainings. This strategy was adopted to gradually build Burera supervisors' capacity to oversee Bandebereho implementation and to lead future trainings. Burera CHW supervisors acted primarily as observers during the couples training and TOT for Cohort 1 but played a more active role in the TOT for Cohort 2. RWAMREC staff were on site at the TOTs to provide coaching and mentorship. Staff from the Ministry of Health, RBC, district officials from Musanze and Burera, and management from the district hospitals were present at different trainings to demonstrate their support.

Immediately after completing the TOT, CHWs finalized the recruitment of eligible men and began facilitating the 17-session curriculum weekly with small groups of up to 12 couples.^1^ All CHWs received kits to support facilitation (e.g., a bag, manual, umbrella, vests, dolls and other materials required for facilitating the activities). After being trained, each pair of CHWs is expected to facilitate two cycles of the curriculum with new participants per year. Given the intensive nature of facilitating Bandebereho, CHWs in the current scale up received 15,000 Rwandan francs (about US$10.55) per cycle facilitated, dispersed in three mobile money payments. The funds were designed to remunerate CHWs for their time and commitment. CHW supervisors in Burera supervised Bandebereho implementation by trained CHWs, with support from CHW cell coordinators, who liaise with the health center and supervise CHWs from the villages within their cell.

### Ethical considerations

Informed consent was obtained from all study participants. CHWs who participated in the short phone survey received 1,000 Rwandan francs (about US$0.70). The funds covered the cost of electricity to charge CHWs' phones, as individuals in Rwanda are not charged airtime to receive mobile calls. The data were collected as part of a larger process and impact evaluation that received ethical approval from the Rwanda National Ethics Committee (RNEC 124/2024) and research permission from the National Council on Science and technology (NCST/482/0080/2024) and Burera district authorities.

### Measures

CHWs completed a 25-item questionnaire prior to the couples training (pre-survey) and a 33-item questionnaire after completing the TOT (post-survey), six (Cohort 1) to nine (Cohort 2) months later. Surveys were completed by all CHWs present and willing on the first and last days of training, respectively. The pre/post surveys assessed CHW attitudes about gender roles and violence pre- and post-training, using statements adapted from the Gender Equitable Men (GEM) Scale (46) and previously tested in Rwanda (34, 47). The questions asked respondents whether they strongly agreed, agreed, disagreed, or strongly disagreed with individual statements (e.g., “It is natural and right that men have more power than women in the family”). Changes in gender attitudes were measured to assess the efficacy of the training, which aims to challenge unequal gender norms and power imbalances between men and women, which perpetuate violence. It is important that CHWs tasked with facilitating Bandebereho support and see the benefits of gender equality, to be able to facilitate changes in participants' gender attitudes and behavior.

The post-survey also asked CHWs their perceptions of the training and whether it equipped them with the knowledge, confidence, or skills needed to implement Bandebereho, their perceived sense of support from community leaders, and knowledge of local violence against women services. These questions were not asked at pre-survey as it was not possible for CHWs to ascertain their readiness to implement prior to engaging with the curriculum. CHWs who participated in the first training with their intimate partner were also asked retrospectively whether they had experienced any changes in their relationship after the couples training, including a sense of partner support for their work as CHWs. Respondents were asked whether they strongly agreed, agreed, disagreed, or strongly disagreed with a series of statements. CHWs who were unable to participate in the couples training or whose partner did not participate were not asked these questions. Pre- and post-surveys were self-completed by CHWs in Kinyarwanda, collected by the study team, and entered in Excel.

The 17-item phone survey asked CHWs about their recent experience implementing the Bandebereho program, eight (Cohort 2) to 13 (Cohort 1) months after completing training. CHWs were asked if they implemented a session in the last two weeks, and if yes, how it went and how comfortable or confident they felt during the session. All CHWs were asked whether they agreed with a series of statements about the impact of the time they spend on Bandebereho on their CHW and family responsibilities. The survey also included several measures not explored in this article: time-use data on Bandebereho implementation and support provided to Bandebereho participants outside of the sessions. The phone survey was administered in Kinyarwanda by trained researchers from an external research firm and data collected in SurveyCTO.

Routine program monitoring data were used to track CHW participation and to assess the quality and fidelity of Bandebereho implementation. Training attendance and session logs tracked CHW participation in training and implementation, respectively. Session intake and attendance logs completed by CHWs captured the date, topic, and duration of each session, and participant demographic and attendance data. CHWs submitted the paper forms to CHW supervisors who entered the data in Excel. Session observation checklists were completed by CHW supervisors and by CHW cell coordinators when visiting Bandebereho sessions facilitated by the CHWs. The checklists assessed implementation on 11 indicators chosen by RWAMREC staff to assess quality and fidelity, using a five-point scale. The indicators included CHW preparedness, professionalism, performance, fidelity to the manual, ability to maintain focus on the topics, group dynamics and participation, women's active participation, and the meeting space, privacy, and seating arrangement. A longer checklist was piloted in the transition-to-scale and simplified for use in the current scale up. The study team collected a sample of completed paper checklists (*n* = 749) from CHW supervisors and entered the data in Excel.

### Analysis

When analyzing the survey data, frequencies were tabulated for the full sample, disaggregated by gender. The pre-survey sample was used as a master sample to create matched datasets with the post-training and phone survey, to compare the results. Pre- and post-survey data were used to present any changes in gender attitudes from pre- to post-training. Post-survey data were examined to understand perceived capacity to implement and perceived changes in partner relations post-training. For most questions, CHWs were asked about the level of agreement on a four-point scale, however, most frequencies presented in this article are merged responses.

Secondly, independent sample t-tests were used to calculate *p*-values to assess if the differences between female and male CHWs' responses were statistically significant. Thirdly, paired sample t-tests were used to compare the responses between pre- and post-surveys among each group of CHWs, female and male. Fourthly, independent sample t-tests were used to examine the differences between Cohort 1 and Cohort 2 on the post-survey and phone survey. Only significant results are described in the text.

Linear regression was used to identify any statistically significant relationship between changes in gender attitudes from pre- to post-survey, with independent variables such as age, gender, education and duration of being a CHW. All CHWs were assigned a score of 1–4, depending on the level of shift in their agreement on each gender-role attitude (from agreement to disagreement, disagreement to agreement, or no shift with agreement at pre-survey and post-survey or disagreement at pre-survey and post-survey). Codes were reversed for an item if needed. The scores for each attitude were added to create a summative score, with a lower score indicating a positive shift in gender attitudes. The analysis has been used to understand which factors are associated with a positive shift in gender attitudes. Analysis was conducted in Stata 17. Routine monitoring data were analyzed in Excel.

## Results

Pre- and post-surveys were completed by 562 and 564 CHWs respectively, with a total of 476 matched pairs with pre- and post-surveys. A total of 506 CHWs completed the phone survey; 34 CHWs were not reached despite multiple attempts; 12 were in the process of being replaced in their roles as CHWs; and three did not consent to participate.

Table 1 presents the pre-survey socio-demographic characteristics of CHWs. The mean age was 38.8 years, with the youngest CHW aged 20 years and the oldest 60. More than half (55.7%) had completed primary school, and more than a third (35.5%) had attended or completed some form of secondary or higher education. The majority (> 80%) had been a CHW for three years or more. Nearly all CHWs were married (98.4%) and had children (99.5%).

**Table 1:** Community health worker socio-demographic characteristics at baseline, *n* = 562.

Variable	Female CHWs	Male CHWs	Total
	*n* (%)	*n* (%)	*n* (%)
Gender distribution of sample	274 (48.8)	288 (51.2)	562 (100)
Age			
Years (mean, SD)	38.4 (7.7)	39.1 (7.2)	38.8 (7.5)
Min, Max	20, 60	23, 60	20, 60
Highest level of education completed			
Some primary school	28 (10.2)	22 (7.6)	50 (8.9)
Primary complete	131 (47.8)	182 (63.2)	313 (55.7)
Some secondary school	61 (22.3)	49 (17.0)	110 (19.6)
Secondary complete	53 (19.3)	34 (11.8)	87 (15.5)
Some university or university complete	1 (0.4)	1 (0.4)	2 (0.36)
Years in current position			
Less than 1 year	27 (9.9)	42 (12.3)	69 (12.3)
1–2 years	28 (10.2)	15 (5.2)	43 (7.6)
3 years or more	219 (79.9)	231 (80.2)	450 (80.1)
Relationship status			
Married	267 (97.5)	286 (99.3)	553 (98.4)
Widowed/Separated/Divorced	7 (2.6)	2 (0.7)	9 (1.6)
Has children	272 (99.3)	287 (99.6)	559 (99.5)

SD, standard deviation.

Table 2 describes CHW reports of preparedness to deliver the Bandebereho program at post-survey (after TOT). Most reported a high level of preparedness, with no significant differences between female and male CHWs. A larger proportion of CHWs “strongly agreed” they had the confidence, clarity of their role, and support of local leaders to implement, while a smaller proportion of CHWs “strongly agreed” they had the skills needed to implement or knowledge of local services for survivors of violence.

**Table 2:** Self-reported capacity and preparedness after completing training, *n* = 564.

Statement	Female CHWs (*n* = 278)		Male CHWs (*N* = 286)		*p*-value for difference between female and male CHWs (strongly agree)
	Agreed or strongly agreed^a^	Strongly agreed^b^	Agreed or strongly agreed	Strongly agreed	
	%	%	%	%	
I have the knowledge needed to implement	98.2	56.1	98.6	54.5	*p* = 0.7084
I have the skills needed to implement	96.4	44.6	95.1	45.1	*p* = 0.9051
I have the confidence needed to implement	99.6	68.4	98.9	67.1	*p* = 0.7587
My role is clear and I know what is expected of me	99.3	68.4	99.3	66.8	*p* = 0.6926
I have the support of local leaders to implement	100	70.1	98.3	70.6	*p* = 0.8997
I am knowledgeable about local services for survivors of violence	97.1	51.4	96.9	54.6	*p* = 0.4608

*t* tests used to assess differences between female and male CHWs.

^a^ This column combines respondents who either “agreed” or “strongly agreed” with a statement, as opposed to those who “disagreed” or “strongly disagreed”.

^b^ This column presents the same item coded differently, presenting only those respondents who “strongly agreed” with a statement.

The post-survey was completed by 299 CHWs from Cohort 1 and 265 from Cohort 2. Analysis by cohort found that Cohort 2 (which received a slightly modified training) was more likely to report positive training outcomes across all six variables compared to Cohort 1 (all statistically significant). For example, 81% of Cohort 2 “strongly agreed” they had the confidence needed to implement, compared to only 56% of Cohort 1 (*p* < 0.001). Similarly, 82% of Cohort 2 “strongly agreed” their role was clear, compared to only 55% from Cohort 1 (*p* < 0.001). Both cohorts were similar in terms of their mean age, marital status, and educational attainment. Cohort 2 had a slightly higher proportion of female than male CHWs than Cohort 1 (52 vs. 47%), while Cohort 1 had a slightly higher proportion of CHWs with ≥3 years of experience than Cohort 2 (82 vs. 78%).

Table 3 compares the gender attitudes of CHWs at pre-survey (before couples training) and six (Cohort 1) to nine (Cohort 2) months later at post-survey (after TOT). The analysis compares changes in attitudes for 476 matched pre- and post-surveys. Female CHWs reported statistically significant changes on 11 out of 12 attitude statements and males on 10 out of 12 statements. Despite female CHWs having slightly more inequitable attitudes at pre-survey, no significant differences between female and male CHWs were seen at post-survey. Further, no significant differences were found between Cohort 1 and Cohort 2, suggesting the training led to comparable changes in attitudes regardless of gender or cohort.

**Table 3:** CHWs' gender attitudes before and after completing full training (*n* = 476).

CHWs who strongly agreed or agreed with the statement^a^	Female CHWs			Male CHWs			*p* value for difference between female and male CHWs^c^
	(*N* = 232)			(*N* = 244)			
Statement	Pre	Post	*p* value^b^	Pre	Post	*p* value	
	%	%		%	%		
A woman's most important role is to take care of her home and cook for her family.	89.9	24.5	*p* < 0.001	78.1	17.7	*p* < 0.001	pre *p* = 0.0595
							post *p* = 0.0632
A man should have the final word about decisions in his home.	34.5	0.9	*p* < 0.001	34	2.1	*p* < 0.001	pre *p* = 0.9149
							post *p* = 0.2831
Sometimes a woman deserves to be beaten.	5.1	0.4	*p* < 0.001	1.2	0.4	*p* = 0.3138	pre *p* = 0.0138
							post *p* = 0.9716
Changing, bathing and feeding the kids are the mother's responsibility, not the fathers.	23.3	2.6	*p* < 0.001	14.7	0.4	*p* < 0.001	pre *p* = 0.0176
							post *p* = 0.0488
It is natural and right that men have more power than women in the family.	53.4	22.8	*p* < 0.001	39.8	22.5	*p* < 0.001	pre *p* = 0.0027
							post *p* = 0.9371
A good woman never questions her husband's decisions, even if she disagrees with him.	27.6	8.2	*p* < 0.001	22.5	6.9	*p* < 0.001	pre *p* = 0.2047
							post *p* = 0.6150
It is a woman's responsibility to avoid getting pregnant.	27.6	7.3	*p* < 0.001	19.7	3.2	*p* < 0.001	pre *p* = 0.0420
							post *p* = 0.0479
A father should be as involved in caring for his children as the mother.	97.4	97.8	*p* = 0.7397	97.5	96.7	*p* = 0.5940	pre *p* = 0.9297
							post *p* = 0.4533
A woman should tolerate violence in order to keep her family together.	11.6	0.43	*p* < 0.001	9.8	1.6	*p* < 0.001	pre *p* = 0.5262
							post *p* = 0.1970
A man should be respected as the head of the household.	90.5	33.6	*p* < 0.001	83.2	23.3	*p* < 0.001	pre *p* = 0.0184
							post *p* = 0.0130
A man must make the final decision on how money is spent in the family.	19.9	0.43	*p* < 0.001	22.1	2.0	*p* < 0.001	pre *p* = 0.5544
							post *p* = 0.1142
A woman must tolerate all the challenges she faces in her household (that's the way of things).	24.6	0.43	*p* < 0.001	17.6	0.4	*p* < 0.001	pre *p* = 0.0632
							post *p* = 0.9716

^a^ Combines respondents who either “agreed” or “strongly agreed” with a statement, as opposed to those who “disagreed” or “strongly disagreed”.

^b^ Paired *t*-test.

^c^ Independent sample *t*-test.

Linear regression analysis showed that those with some secondary education were significantly less likely to hold inequitable attitudes post-training compared to those with some primary or no formal schooling (coefficient: −2.15, *p* < 0.01), controlling for age, gender and duration of being a CHW. These CHWs either did not hold inequitable attitudes prior to training or shifted toward holding more equitable attitudes after training. Further, those who had been CHWs for longer (≥3 years) were less likely to hold inequitable attitudes compared to those with less experience (coefficient: −2.27, *p* < 0.001). This pattern was seen in both cohorts.

Table 4 describes relationship changes reported by CHWs at post-survey. A majority of CHWs agreed the couples training led to relationship improvements, with the largest proportion agreeing their partner was more supportive of their role as a CHW. A higher proportion of male CHWs reported relationship improvements compared to females. This difference appears to be driven by Cohort 1, as no statistically significant differences between female and male CHWs were found in Cohort 2. The second cohort also reported a higher level of agreement on all aspects of partner relations compared to the first cohort. For instance, 82% of Cohort 2 strongly agreed that “my partner and I discuss household decisions often”, compared to 70% of Cohort 2. Similarly, 89% of Cohort 2 “strongly agreed” that “my partner and I better manage childcare and household responsibilities” compared to 72% of Cohort 1.

**Table 4:** CHW reports of relationship changes six to nine months after the couples training (*n* = 518).

Reports of CHWs who participated in training with their partners (*n* = 518)	Female CHWs		Male CHWs		*p* value for difference between female and male CHWs (strongly agreed)
	(*N* = 247)		(*N* = 271)		
Statement	Agreed or strongly agreed^a^	Strongly agreed^b^	Agreed or strongly agreed	Strongly agreed	
	%	%	%	%	
My relationship with my partner has become stronger.	99.2	73.7	99.6	83.4	*p* = 0.0069
It has become easier to communicate with my partner.	98.8	68.4	99.3	80.1	*p* = 0.0023
My partner and I argue less often.	97.2	69.6	99.6	78.2	*p* = 0.2055
My partner is more supportive or encouraging of my role as a community health worker.	99.6	83	99.3	88.9	*p* = 0.0230
My partner and I discuss household decisions more often.	98.4	71.3	99.6	79.3	*p* = 0.0162
My partner and I better manage childcare and household responsibilities between ourselves.	98.8	73.7	99.3	84.9	*p* = 0.0049

^a^ This column combines respondents who either “agreed” or “strongly agreed” with a statement, as opposed to those who “disagreed” or “strongly disagreed”.

^b^ This column presents the same item coded differently, presenting only those respondents who “strongly agreed” with a statement.

Table 5 presents key variables from the phone survey on CHW experiences and perceptions of implementing Bandebereho (eight to 13 months after completing training). Eighty-three percent of CHWs had facilitated a Bandebereho session in the last two weeks. The majority of these CHWs reported a high degree of overall session performance, comfort, and confidence, although male CHWs were more likely to report feeling “very confident” than females. Analysis by cohort found that while a higher proportion of Cohort 2 reported feeling “very comfortable” and “very confident” compared to Cohort 1; these differences were not statistically significant. Further, the majority of CHWs “strongly agreed” the time spent on Bandebereho enriches their community health work and “strongly agreed” that it strengthens their relationships with their partner and family. However, a small proportion of CHWs “strongly agreed” or “agreed” the time spent on Bandebereho made it difficult for them to perform their other CHW responsibilities, or their family or household responsibilities. There were no statistically significant differences between female and male CHWs. Further, there were no significant differences by cohort, with the exception of those who agreed the time spent makes it difficult for them to perform their other CHW responsibilities (13% of Cohort 1 agreed vs. 6% of Cohort 2 (*p* < 0.01).

**Table 5:** CHW experiences of implementing bandebereho 8–13 months after completing training (*n* = 506).

Variable	Female CHWs	Male CHWs	*p*-value for difference between male and female CHWs
	(*n* = 256)	(*n* = 250)	
	*n* (%)	*n* (%)	
Facilitated a session in the last two weeks	212 (82.8)	206 (82.4)	
How the session went	*N* = 212	*N* = 206	
Very well	60 (28.3)	65 (31.6)	*p* = 0.4691
Well	149 (70.3)	137 (66.5)	
Poorly	3 (1.4)	0 (0)	
Confidence level			
Very confident	124 (58.5)	144 (69.9)	*p* = 0.0150
Confident	86 (40.6)	62 (30.1)	
Not very confident	2 (0.94)		
Comfort level			
Very comfortable	144 (67.9)	135 (65.1)	*p* = 0.5345
Somewhat comfortable	65 (30.7)	69 (33.5)	
Not very comfortable	3 (1.42)	3 (1.5)	
The time I spend on Bandebereho…			
Enriches or improves my confidence as a community health worker	*n* = 256	*n* = 250	
Strongly agree	212 (82.8)	211 (84.4)	*p* = 0.3235
Agree	43 (16.8)	39 (15.6)	
Disagree	1 (0.4)	0 (0)	
Strongly disagree	0 (0)	0 (0)	
Helps strengthen my relationships with my partner and my family			
Strongly agree	203 (79.3)	212 (84.8)	*p* = 0.9699
Agree	48 (18.8)	33 (13.2)	
Disagree	4 (1.6)	3 (1.2)	
Strongly disagree	1 (0.4)	2 (0.8)	
Makes it difficult to perform my other CHW responsibilities			
Strongly agree	5 (1.96)	4 (1.6)	*p* = 0.6035
Agree	21 (8.20)	18 (7.2)	
Disagree	119 (46.5)	107 (42.8)	
Strongly disagree	111 (43.4)	121 (48.4)	
Makes it difficult to perform my family or household responsibilities			
Strongly agree	7 (2.7)	3 (1.2)	*p* = 0.6914
Agree	14 (5.5)	20 (8.0)	
Disagree	119 (46.5)	102 (40.8)	
Strongly disagree	116 (45.3)	125 (50.0)	

*t*-test, independent sample; compares female and male respondents who responded they were “very confident”, “very comfortable”, session went “very well,” or “strongly agree.”

During the study period, CHWs facilitated the first cycle of Bandebereho with 6,655 parents. Analysis of routine program monitoring data found that CHWs demonstrated a high degree of fidelity and quality in the first cycle. Analysis of session attendance logs from all 276 Bandebereho groups found that CHWs delivered the sessions in order according to the curriculum and within the recommended two to three hours (average of 2.21 hours). Data analyzed from a sample of 749 session observation checklists found that CHWs averaged between 4.4 to 4.7 on a five-point scale (5 being the highest) across all 11 indicators, including preparedness (4.6), fidelity to manual (4.4), level of participant interaction (4.5), and ensuring women's voices (4.4). Participant attendance and retention was also strong: men attended on average 13.6 (out of 17) sessions and women 7.9 (out of 10), with a dropout rate of 5.3%. Men who dropped out attended 4.5 out of 17 sessions (26%) on average. At the time of the phone survey, CHWs were implementing a second cycle with 6,504 new parents, which had similar attendance rates and a dropout rate of 4.6%.

## Discussion

Our findings indicate the training assessed in the study equipped CHWs to deliver the Bandebereho program with a high degree of fidelity and quality, which are critical for achieving reductions in violence when delivered at scale. The two-part training included a couples training for CHWs and their intimate partners, followed by a TOT for CHWs on how to facilitate the Bandebereho curriculum. Importantly, staff from the health system co-facilitated the couples training and led the TOT, supported by RWAMREC staff. This represented an important step in the gradual process of shifting responsibility for program implementation to the health system. Bringing experienced CHW supervisors from Musanze district to conduct the training alongside their counterparts in Burera also facilitated a transfer of knowledge between districts. Burera CHW supervisors were able to learn from RWAMREC and from their peers in Musanze, who have been involved in implementing Bandebereho since 2019. This knowledge transfer is now being extended to a third district, where Burera supervisors are leading the training of CHWs, supported by their peers in Gakenke district.

The findings also indicate the training modifications made between the first and second cohort increased the efficacy of the TOT. The second cohort reported a greater sense of having the necessary skills, knowledge, and confidence to implement Bandebereho compared to the first cohort. The additional time for CHWs to practice facilitating the gender transformative curriculum likely enhanced the second cohort's sense of preparedness to implement. The findings also suggest the second cohort benefited from meeting local leaders earlier than their peers in the first cohort. The second cohort reported a greater sense of support from local leaders, which research suggests may support their retention, motivation, and performance (48, 49). Indeed, RWAMREC staff observed the important role of local leaders in motivating CHWs and supporting them to recruit and retain group members. By the time of the phone survey (during the second cycle of implementation) we found no differences between cohorts in terms of their self-reported confidence, comfort, or performance. This suggests the experience of facilitating the first cycle of Bandebereho sessions (“learning by doing”) increased CHWs' capacity to facilitate the participatory and interactive sessions.

The Bandebereho trainings also led to more equitable gender attitudes among CHWs over a six-to-nine-month period. Importantly, both male and female CHWs reported more equitable attitudes after training, despite female CHWs demonstrating less equitable attitudes initially. Other research in Rwanda has also found less equitable gender attitudes among women compared to men, including greater acceptance of violence against women (29). Also consistent with findings in the literature, we found that CHWs with more education displayed more equitable attitudes after training (50, 51). The shifts we found toward more equitable attitudes about gender roles matter because CHWs operate within the same gendered systems as the broader community (52, 53). Promoting critical reflection on gender norms and unequal power dynamics, a core component of many violence prevention programs, is difficult if facilitators have not bought into gender equality (4, 43). Facilitators who believe in the benefits of more equitable roles and relationships are likely to be more effective at changing the attitudes and behavior of others. Indeed, the Becoming One program in Uganda found that facilitators with more equitable attitudes were associated with greater reductions in IPV among participants (54). Despite the positive shifts we found, a considerable proportion (22% to 30%) of CHWs in our study still agreed that a woman's most important role is to take care of her family, that it is natural for men to have more power than women, or that men should be respected as heads of household. Transforming these more deeply entrenched attitudes about gender roles, which are tied to hierarchies of power, may require longer-term and more structural social changes (34).

The findings also affirm the importance of the couples training as a core component of building CHW capacity to deliver Bandebereho. As a result of the training, CHWs reported stronger relationships, less arguing, easier communication, more discussion of household decisions, and better management of household responsibilities with their partners. Across the full sample, male CHWs were more likely to report relationship improvements than females, although this was driven by gendered differences in the first cohort. Relationship changes like these often take a longer period to become measurable (21), as it takes time for couples to adopt new behaviors and begin to see benefits, which can help reinforce or sustain these changes. CHWs in the second cohort had nine months to adopt these new behaviors, while the first cohort had only six. Male CHWs in the first cohort probably found it easier to adopt these new behaviors compared to their female peers—as they require changes in *men's* behavior. The Bandebereho program asks men to build more equitable relations with their partners by sharing power in household decisions and by taking on their fair share of the unpaid care work, which often falls solely on women's shoulders. This is critical in a context where women spend a disproportionate amount of time on unpaid care work compared to men, despite also performing paid work (55). The couples training also enabled CHWs to experience the Bandebereho program before facilitating it with others. Being able to personally speak to the program's benefits may foster CHWs' confidence and motivate them to support others in adopting more caring, supportive and non-violent relationships.

The positive relationship changes reported are important for CHWs' own wellbeing but may also make it easier for them to provide critical health services. Studies in numerous settings have identified family disapproval and a lack of partner support as barriers to the work of CHWs, especially females (56, 57). Six to nine months after the couples training, the majority of female (83%) and male (89%) CHWs in our study “strongly agreed” their partner was more supportive or encouraging of their role as a CHW. Nearly one year later, the majority of CHWs “strongly agreed” the time they spend on Bandebereho enriches their relationships with their partners and family (82%) and their confidence as CHWs (84%). However, a small proportion (less than 10%) “agreed” or “strongly agreed” the time spent on Bandebereho made it difficult for them to manage their CHW or family responsibilities. Previous research with CHWs in Rwanda has found the varied and unpredictable nature of the work detracted from the time necessary for families (49, 58). Research in other settings has found that female CHWs in particular may struggle to balance their work and family responsibilities, given gendered expectations that women perform most of the unpaid care work (56, 57). Critically, male CHWs in our study were as likely as females to report such a time burden, suggesting that involvement in Bandebereho did not exacerbate existing gender disparities. However, the findings deserve further consideration in light of concerns that CHWs and other frontline workers are already overburdened (59–61).

Our findings underscore the importance of investing in high quality training, which includes adequate time to support facilitators' personal transformation, when taking programs to scale. Facilitators who have never been exposed to gender transformative programming need time to reflect on their own values and biases, to understand gender, learn the curriculum, and build and practice participatory facilitation skills (43). Most effective violence prevention programs include at least ten days of training, and it has been suggested that as many as 25 days may be needed to build facilitators' capacity (4, 43). Yet, ten days of training is often seen as unrealistic at scale, and training time is often decreased and space for critical reflection removed amidst pressure to reduce program time and cost. To achieve this, cascade training models are often used to train facilitators at scale, where experts train a small group of trainers who then train others, with the cycle cascading down until the last group is trained. This model can significantly reduce training quality and efficacy, particularly for complex gender transformative programs, further limiting the potential to change facilitators' attitudes (43). The challenges of cascade training models have also been documented when scaling maternal and child health programs with CHWs (62). RWAMREC deliberately avoided a multi-level cascade training model. Instead, they invested in high quality training and supervision, progressively built health system capacity, and gradually tapered their assistance, an approach which seems to have paid off.

The findings also suggest there is still scope to improve the training for CHWs. The time between the couples training and TOT was far too long, necessitating refresher training which increased training time and cost. Several factors contributed to these delays, some of which were outside the control of RWAMREC staff. Better sequencing of the two trainings in future can potentially reduce the overall training time (and cost) without compromising quality. If delivered closely together, the two trainings can build upon each other synergistically, averting the need to refresh CHWs' knowledge of material covered in the first training. This can also reduce the time between initiating training and starting to implement the program with couples in the community. The findings also suggest more can be done to build CHWs' knowledge of local services where they can refer survivors of violence.

Our study is not without limitations. Our data rely on self-reported perceptions and changes in behavior, rather than direct observation. Social desirability bias may have impacted CHWs' responses to the survey questions, although the findings are similar to observations made by RWAMREC staff. We were only able to gather data on CHWs' relationships at one time point, asking them to retrospectively report on changes. The findings also cannot be generalized to all CHWs in Rwanda. Nevertheless, the sizeable sample and the fact that we collected data at multiple points over 20 months helps us to understand the training impact and efficacy. Ongoing qualitative research will provide a more nuanced understanding of CHWs' experiences of Bandebereho training and implementation, including challenges faced. Analysis of the time-use data collected in the phone survey will also help us understand the time required to deliver the program, and for which CHWs the time required represents a significant burden.

The study is novel because Bandebereho is one of few gender transformative programs with proven impact on IPV that is being scaled with government, especially through the health system. Our findings contribute towards better understanding of what it takes to build the capacity of CHWs, or other frontline workers, and the systems around them to deliver effective prevention programming at scale. Future research should explore whether Bandebereho, and similar programs in other settings, continue to achieve reductions in IPV and other outcomes when delivered at scale. Additional evidence on the relationship between implementation fidelity and quality and program outcomes would also support practitioners in scaling these approaches. Lastly, future research should explore whether and how these programs contribute to broader systems change when scaled with government.

## Conclusion

The Bandebereho experience indicates it is feasible to equip an existing government workforce to deliver a gender transformative IPV prevention program at scale. Our findings highlight the importance of investing in facilitator training to ensure implementation fidelity and quality at scale. By far, training CHWs and their supervisors is the largest cost of the Bandebereho scale up. Yet, this early investment in capacity building in the first year in a new district can ultimately enable the health system to deliver Bandebereho at relatively low-cost thereafter. Once trained, the CHWs in Burera district can reach more than 12,000 parents per year, allowing for exponential growth as the program expands to new districts (13, 15). In addition to reducing IPV and violence against children, Bandebereho improves maternal health-seeking, family planning uptake, and child development, among other outcomes, and helps to build health workforce capacity. Thus, Bandebereho presents a relatively cost-effective way for the Rwandan government to achieve multiple of its health and development goals, including increasing antenatal care attendance and reducing IPV. At the time of writing, more than 1,000 CHWs trained in Musanze and Burera districts have reached over 45,000 parents, and rollout is ongoing in a third district.

Our findings also underscore the importance of a slow and steady approach, which allows sufficient time to adapt, test, and refine the program model for scale. The phased approach allowed RWAMREC to work hand-in-hand with government to test program modifications, generate learning, and make improvements between districts. This allowed time to adapt the training, demonstrate to the health system what high-quality implementation looked like, and progressively hand over responsibility. As a field, we can do more to demonstrate the important return on investment of such an approach and highlight the risks that inadequate training or scaling too quickly pose—including limited impact, backlash, or even a rise in violence (6, 15, 39, 63). These risks undermine the very reason for taking programs to scale and go against the “do no harm” principles of gender transformative programs (8, 63). As others have pointed out, scaling with government requires time and strong partnerships (9, 12), and it may cost more than has traditionally been assumed (64). We hope our findings provide insights for other organizations and funders interested in scaling IPV prevention programs through public systems in other settings.

## Data Availability

The raw data supporting the conclusions of this article will be made available by the authors, without undue reservation.

## Footnotes

^1^The Bandebereho program recruits couples via the male partner. Eligible men must: be an expectant or current parent of a child under five years; be married/cohabiting with a partner for at least three months; live in the local community; be 21 to 40 years; and be committed to participate.
